# Improved visualisation of hepatic metastases in gadoxetate disodium-enhanced MRI: Potential of contrast-optimised (phase-sensitive) inversion recovery imaging

**DOI:** 10.1371/journal.pone.0213408

**Published:** 2019-03-06

**Authors:** Ute Lina Fahlenkamp, Günther Engel, Lisa Christine Adams, Sarah Maria Böker, Minh Huynh Anh, Moritz Wagner, Bernd Hamm, Marcus Richard Makowski

**Affiliations:** Department of Radiology, Charité—Universitätsmedizin Berlin, Berlin, Germany; Medical University of Vienna, AUSTRIA

## Abstract

**Background:**

Detection of metastases can have a significant impact on therapy. Nevertheless, even in gadoxetate disodium-enhanced MR scans, very small hepatic metastases may be difficult to see.

**Purpose:**

To investigate the potential of a contrast-optimised (phase-sensitive) inversion recovery MR sequence in gadoxetate disodium-enhanced scans for detection of hepatic metastases.

**Materials and methods:**

With institutional review board approval and after written informed consent, 40 patients (18 male, 22 female) with suspected or known hepatic metastases were examined on a 1.5 T MR system. A T1-weighted gradient-echo volumetric-interpolated-breath-hold (VIBE) sequence was acquired as part of the standard imaging protocol 20 minutes after administration of gadoxetate disodium. Additionally, an IR sequence was acquired with an inversion time to suppress native signal from metastases. Overall image quality and delineation of lesions were assessed on VIBE as well as on magnitude-reconstructed (MAG) and phase-sensitive IR (PSIR) sequences. Lesion-to-liver contrast (LLC) was compared between VIBE and MAG images.

**Results:**

Overall image quality was high in both VIBE and MAG IR sequences (VIBE 4.275; MAG 4.313), yet significantly lower in PSIR (4.038). Subjective delineation of lesions was higher on MAG and PSIR images compared to VIBE in all size groups with an overall statistically significant difference for VIBE vs. MAG vs. PSIR (p < .001) in the variance analysis. Mean LLC was 0.35±0.01 for VIBE sequences, and 0.73±0.01 for MAG.

**Conclusion:**

Contrast-optimised PSIR seems to improve imaging characteristics of hepatic metastases in gadoxetate disodium-enhanced scans compared to T1 gradient-echo VIBE sequences.

## Introduction

Due to its rich blood supply, the liver is one of the organs most frequently affected by metastatic disease. Reliable detection of metastatic disease is of high importance, as tumour treatment may be significantly different depending on absence or presence of metastases. Since its approval in Europe and Asia in 2005 and in the United States in 2008, gadoxetate disodium (Gd-EOB-DTPA) has become well established in hepatobiliary imaging due to its property to be taken up by hepatocytes as they express an organic anion transporting peptide. Lesions without the organic anion transporting peptide do not accumulate gadoxetate disodium and therefore appear hypointense in the hepatobiliary phase compared to the enhancing normal liver parenchyma, improving detection rates of liver metastases [1–3].

In the clinical setting, a frequently used sequence is the gradient-echo T1 breath-hold sequence [4–6]. However, contrast between metastases and surrounding gadoxetate disodium accumulating liver parenchyma is not maximal with this sequence, as a certain positive baseline T1 signal is inherent to most types of metastases, irrespective of the administration of a contrast agent.

Different MR sequence techniques can be used to improve contrast between areas with contrast agent accumulation and surrounding tissues, which has been a focus of interest especially in the context of myocardial imaging: Specific inversion recovery (IR) sequences were developed to minimise the signal from normal myocardium, highlighting the signal from the contrast agent, e.g. in myocardial fibrosis or scar tissue [7–11]. Phase-sensitive inversion recovery (PSIR) imaging represents a clinically robust sequence design and is therefore nowadays included in most cardiac imaging protocols [12]. This sequence technique has the potential to improve the visualisation of liver metastases.

The purpose of this study was to prospectively test the potential of a contrast-optimised PSIR MR imaging technique for the detection of hepatic metastases, compared to a conventional T1 gradient-echo VIBE sequence.

## Material and methods

### Study population

This prospective study was approved by and registered with the local ethics committee (Ethikkommission, Ethikausschuss 1 am Campus Charité –Mitte). From January 2017 to January 2018, patients with known metastases of the liver or a primary tumour known for possible hepatic metastatic spread were referred for MR examination of the liver. Patients provided written informed consent to participate in the study. Exclusion criteria were age younger than 18 years, pregnancy, metallic implants or functional devices not eligible for MR examination, claustrophobia, a history of allergic reaction to Gd-EOB-DTPA, and a glomerular filtration rate below 30 ml/min. According to the eligibility criteria, 40 patients (18 male and 22 female; age range 29–80 years; 57.6 ± 13.1 years; mean ± standard deviation, SD) were included in the study. Primary tumours were breast cancer (n = 14), cancer of the biliary system (n = 2), neuroendocrine cancer (n = 5), cancer of the prostate (n = 1), cancer of the gastrointestinal tract (n = 5), cancer of the thyroid gland (n = 1), renal cell carcinoma (n = 1), angiosarcoma (n = 1), extra gonadal tumour (n = 1), and melanoma (n = 4). Five patients had no known primary tumour, but were referred to MR imaging as preceding examinations (ultrasound or CT) for different reasons resulted in unequivocal hepatic findings.

None of the patients suffered from relevant cirrhosis. On precontrast T1 imaging, metastatic lesions were either isointense or hypointense to liver parenchyma. Previously locally treated lesions were not included in the evaluation.

All included patients underwent standard liver MR imaging using the hepatocyte-specific contrast agent gadoxetate disodium (Gd-EOB-DTPA, Primovist) and additional PSIR sequences.

### Imaging protocol

MR imaging was performed on a 1.5 T scanner (Avanto, Siemens Healthineers, Erlangen, Germany) equipped with a 32-channel body-phased-array coil. Patients underwent the local standard liver MR imaging protocol using gadoxetate disodium which includes an axial T1-weighted spin-echo (SE) sequence, an axial fat-saturated (FS) respiratory-gated T2-weighted turbo SE sequence, an axial T1-weighted dual echo sequence for in-phase and opposed phase imaging, and axial T1 VIBE (volume-interpolated breath-hold) sequences for dynamic imaging before and 15, 55 seconds and 2, 5, 10 and 20 minutes after contrast agent administration and a coronally orientated T1 VIBE sequence for the hepatobiliary phase at least 20 minutes after contrast agent administration. Gadoxetate disodium was manually injected and followed by a 10 ml saline flush.

In addition, after completion of the axial T1 VIBE in the hepatobiliary phase and before acquisition of the coronally orientated, a trueFISP (true fast imaging with steady precession) IR gradient echo sequence was performed and reconstructed using magnitude (MAG) and phase-sensitive (PSIR) reconstruction. In the first three patients (n = 3), investigated in this study, who were suffering from breast cancer, cancer of the colon and gall bladder carcinoma, a TI (inversion time) scout sequence of the liver was performed. The optimal TI for a maximised contrast between hepatic metastases and liver parenchyma was measured and determined to be 480 ± 15 ms leading to full suppression of signal from hepatic metastases and was chosen for all subsequent patients. Spatial resolution and slice thickness were matched to the VIBE sequence. Imaging parameters of the axially acquired IR sequence were as follows: field of view (FOV) 400 x 400 mm^2^, matrix 320, TR (repetition time)/TE (echo time) = 601.22/1.36 milliseconds (ms), 40 degree flip angle, and 3 mm slice thickness. Acquisition time was 21 seconds for T1 VIBE fs, and 23 seconds for the IR sequence. Following the acquisition, IR magnitude and phase images were reconstructed. The imaging parameters for the entire liver imaging protocol are shown in Table 1.

**Table 1:** MR imaging parameters. The table represents of the most important sequence parameters of the standard liver imaging protocol as well as of the (phase-sensitive) inversion recovery sequence. To directly compare VIBE and IR sequences, parameters such as FOV, image matrix and slice thickness were identical for both sequences.

Sequence parameters	T1 FLASH	T1 FLASH double-echo	T2 TSE with PACE	T1 VIBE	(Phase sensitive) inversion recovery
**TR (ms)**	186	177	2430	4.7	601.2
**TE (ms)**	4.76	2.4/5.1	79.0	2.4	1.4
**Flip angle (°)**	70	70	180	10	40
**Inversion time (ms)**	--	--	---	--	480
**Field of View (mm)**	400	400	400	400	400
**Image matrix**	320	256	320	320	320
**Slice thickness (mm)**	4	4	4	3	3
**Acquisition time (s)**				21	23

### Image analysis

All standard liver imaging sequences and IR images were analysed on standard workstations (Centricity PACS, Radiology RA1000, General Electrics).

### Qualitative evaluation

The review was done by two radiologists with six (U.L.F.) and two (G.E.) years of experience in reading abdominal MRI.

General image quality and presence of artefacts were rated on a five-point evaluation scale as follows: 5, excellent, uniform contrast over the entire FOV, no artefacts; 4, good, mild artefacts, no impairment of image interpretation; 3, moderate, artefacts interfering with image interpretation; 2, poor, prominent artefacts, diagnostic quality questionable; and 1, non-diagnostic.

Additionally, subjective impression on contrast of lesions and their delineation was evaluated, stratified by size classes (> 5 cm; 2.5–5 cm; 1–2.5 cm; 0.5–1 cm; < 0.5 cm) using the following five-point evaluation scale: 5, circumferentially clear delineation of the lesion, high contrast to surrounding tissue; 4, slightly reduced sharpness of lesion contours or reduced contrast; 3, slightly reduced sharpness of the lesion contours and reduced contrast; 2, prominently reduced sharpness of the lesion and reduced contrast; 1, lesion hardly to delineate. If several lesions were present in one size group, the lesion to be measured was chosen according to criteria of highest reproducibility (e.g. minimal affection by artefacts on both sequences, homogeneous appearance, largest size possible).

For general image quality and presence of artefacts and subjective impression on contrast of lesions and their delineation, evaluation and comparison to VIBE images were done on MAG as well as on PSIR images in different reading sessions blinded for the results from the other reading session.

### Quantitative evaluation

#### Size measurements

For every size group as defined above, if present, one lesion was chosen and measured on both sequences, which was done by both readers independently from the other. To avoid statistical errors, lesions were only recorded if measurements led to the same size class on both sequences. The same approach was chosen for signal measurements of lesions.

#### Signal measurements

For the assessment of the objective lesion-to-liver contrast, every reader independently placed a circular region of interest (ROI) in the metastases and in the adjacent liver parenchyma, both on VIBE and MAG images. The ROI was chosen manually as large as possible avoiding healthy liver parenchyma, vessels or other structures. Care was taken to avoid interference by artefacts, nevertheless, in very small lesions an interference with partial-volume-effects was conceded. Consistent positioning of the regions of interest across different series was achieved by careful examination of the landmarks (liver contours, spleen contours, etc.) on the images.

Subsequently, the contrast ratio between liver parenchyma and liver lesion (LLC) was calculated as follows:

LLC=(SIliver−SIlesion)/SIliver),

where signal intensity of the liver parenchyma is SI_liver_ and the corresponding signal intensity of the liver lesion is SI_lesion_.

### Lesion-per-lesion analysis

Finally, a direct comparison of lesions (lesion-per-lesion analysis) visualised on VIBE and on IR images was undertaken in a consensus reading by two radiologists with six (U.L.F.) and nine (M.R.M.) years of diagnostic experience in hepatobiliary imaging: On VIBE images, metastases were identified if a lesion with low signal intensity relative to the surrounding liver parenchyma corresponded to a lesion with moderately hyperintense signal on the T2 FS sequence. Metastases on IR images were classified as such if a lesion of very low signal intensity relative to the surrounding liver parenchyma, usually with a sharp demarcation, corresponded to a lesion of moderately hyperintense signal on the T2 FS sequence. Evaluation of IR images was undertaken by taking into account both magnitude-reconstructed and phase-sensitive images.

### Statistical analysis

Interrater reliabilities were assessed by calculating interclass correlation coefficients (ICC). Each calculation was based on a mean-rating, absolute-agreement, 2-way mixed-effects model. Values of less than.5 were considered to be indicative of poor reliability, values between.5 and.75 were taken to indicate moderate reliability, values between.75 and.9 signified good reliability, and values greater than.90 indicated excellent reliability.

Regarding subjective contrast, LCC and size, a number of calculations were conducted. First, interrater agreements were evaluated at the level of individual sizings. Then, ICC mean-ratings were calculated involving the pooling of all measurements, irrespective of size.

Following the assessment of interrater reliabilities, the data of both raters were merged and a number of one-way analyses of variance with repeated measures were conducted.

With regards to image quality, a one-way repeated measures ANOVA was conducted.

Regarding the investigation of possible differences at the levels of subjective contrast, LLC and size a number of ANOVAs were conducted. First, differences were evaluated at the levels of individual sizing. Subsequently, the data collected for all sizes were pooled and an additional ANOVA was conducted comparing mean scores across all sizes.

For each model, a p-value of < .05 was regarded as indicative of a statistically significant difference. In those instances where a model involved more than two levels of repeated measures, i.e. VIBE, MAG as well as PSIR, additional pairwise comparisons using the Bonferroni correction were conducted.

## Results

### Qualitative evaluation

**Fig 1:**
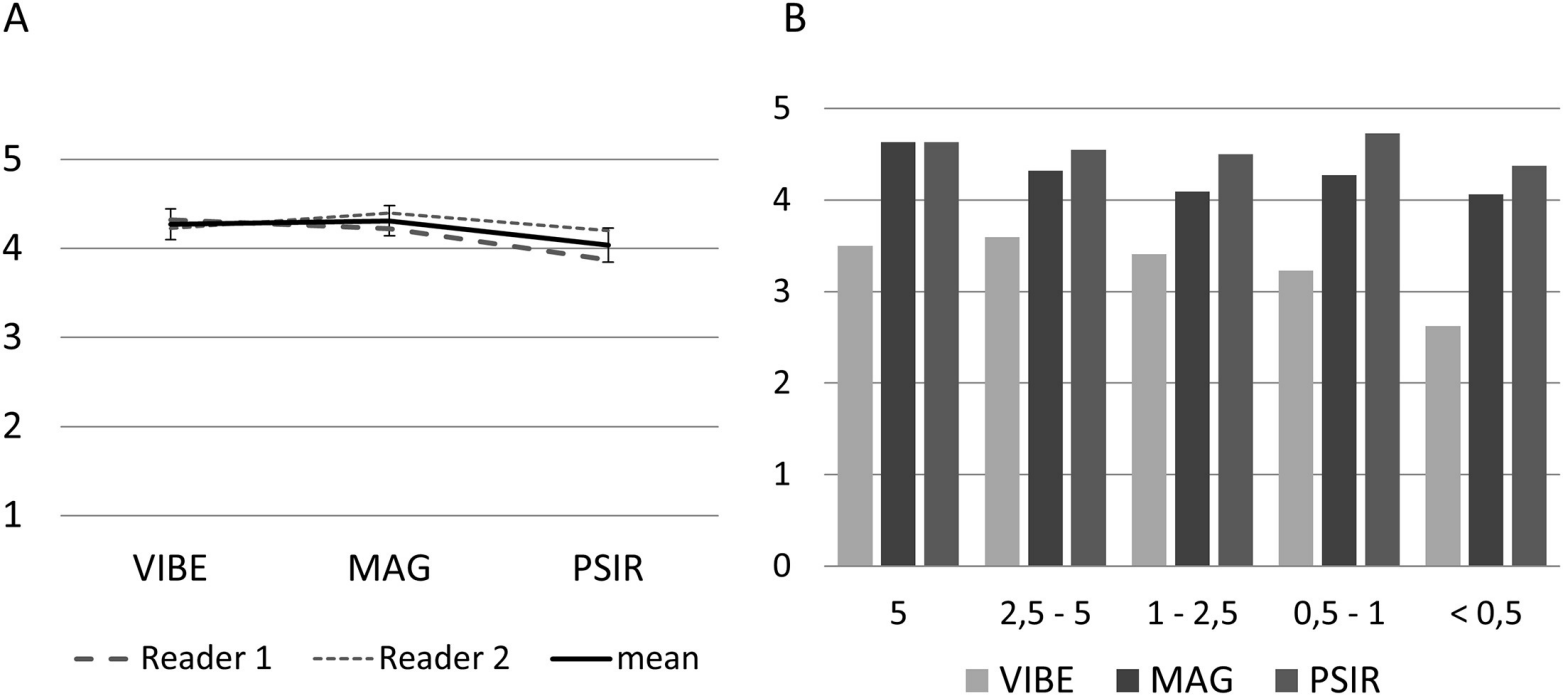
Overall image quality and subjective delineation and visual contrast. (A) Overall image quality as rated by readers and mean. Whiskers indicate 95% confidential interval. (B) Subjective delineation and visual contrast. The score describing subjective delineation and visual contrast was higher on MAG and PSIR sequences compared to VIBE in all size groups; overall, there was a statistically significant difference for VIBE vs. MAG vs. PSIR (p < .001).

For overall image quality, ICC mean-ratings were found to be moderate to good with values ranging from.575 to.805. Overall image quality as an average of both readers was high in both VIBE and magnitude-reconstructed IR sequences (VIBE estimated marginal mean: 4.275; MAG estimated marginal mean: 4.313) and significantly lower in phase-sensitive reconstructed IR (PSIR estimated marginal mean: 4.038; VIBE vs. PSIR 0.015 and MAG vs. PSIR p < 0.001) (Fig 1(A)). Main factors contributing to impaired image quality on VIBE sequences were increased noise in the central parts of the body distant to the coil position and motion-artifacts, whereas impaired image quality on IR sequences were mainly due to B1 field inhomogeneity.

For subjective contrast, ICC mean-ratings on an individual size level were largely found to be moderate to excellent with values ranging from.526 to.926, yet comparisons for both size.05–1 and size < .05 were found to be of insufficient reliability, with each showing a mean-rating of.000. For the model that involved the pooling of all measures, the ICC mean-ratings were found to be moderate to good with values ranging from.516 to.851.

**Fig 2:**
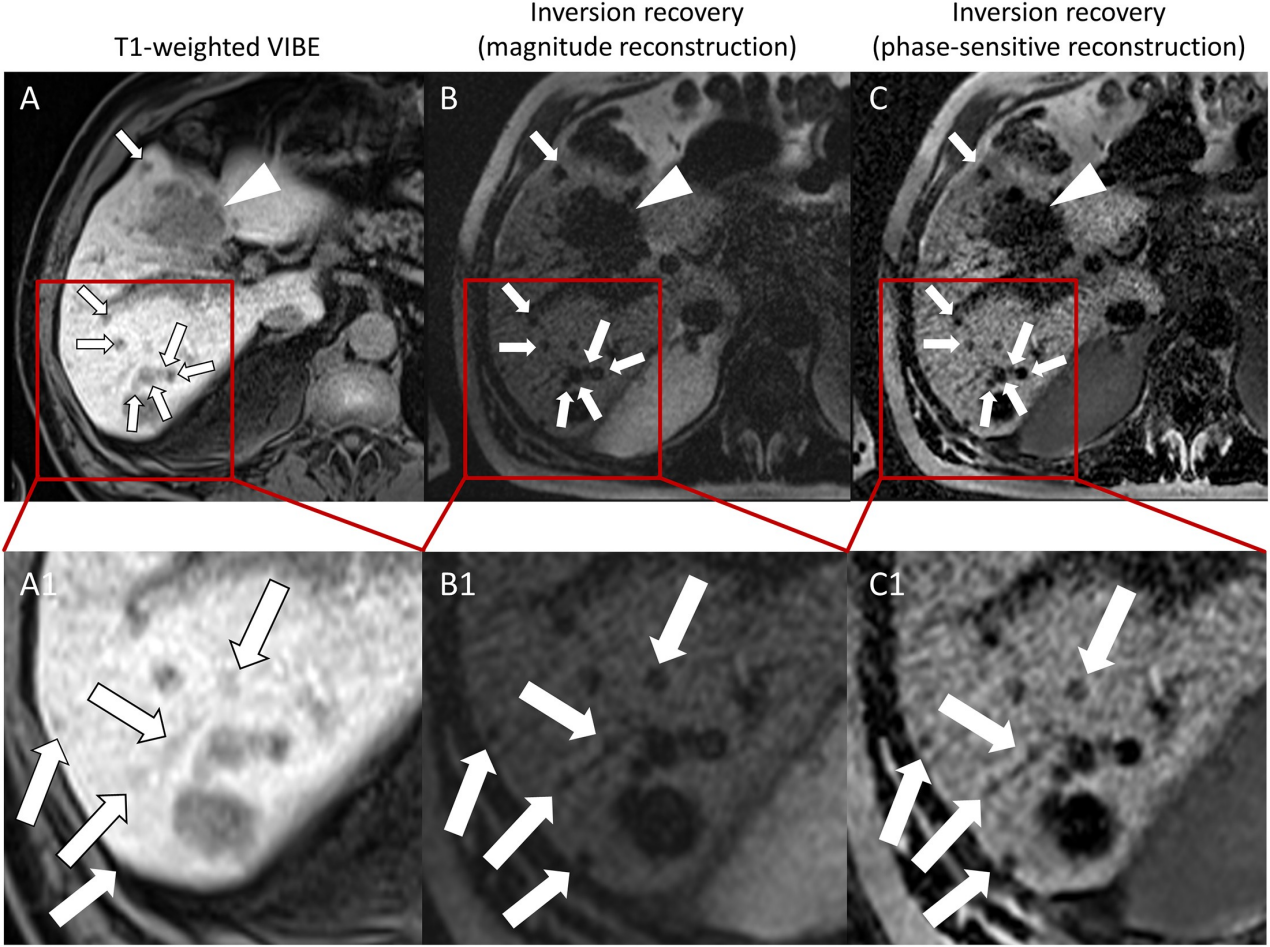
Axial images in a 74-year-old man with gall bladder carcinoma acquired with (A) T1 VIBE (repetition time msec/echo time msec, 4.74/2.38), and (B,C) T1-weighted inversion recovery (912/1.13) by using (B) magnitude and (C) phase-sensitive reconstruction 20 minutes after administration of gadoxetate disodium. Note the distinct conspicuity of the lesion in segment VII (arrowhead) and the clear delineation of the small lesions (white arrows). Five lesions which are distinctly delineated on inversion-recovery images (B1, C1) are hard to detect on VIBE (A1), which illustrates the high relative liver-to-lesion contrast.

**Fig 3:**
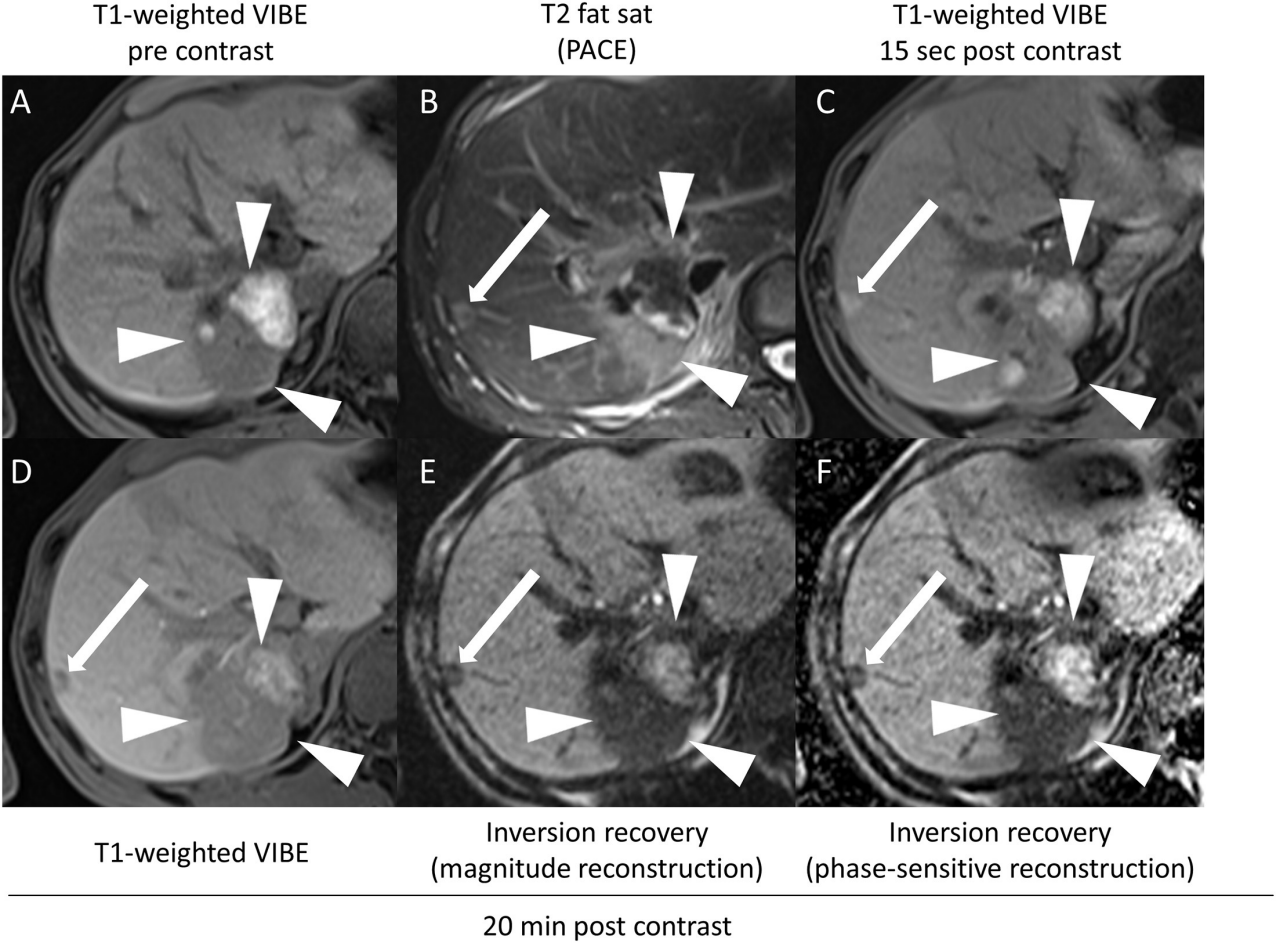
Images of a 61-year-old man with hepatic metastases of malignant melanoma. Constant appearance of a large haemorrhagic lesion (white arrowheads) that had been treated by local ablation beforehand. Nevertheless, a new metastatic lesion (white arrow) was seen in segment VIII: compared to the VIBE sequence (d), the lesion is more sharply demarcated on PSIR images with magnitude reconstruction (e), as well as with phase-sensitive reconstruction (f).

The score describing subjective delineation and visual contrast was higher on MAG and PSIR sequences compared to VIBE in all size groups; mean score in metastases larger than 5 cm was 4.63±0.52 (MAG and PSIR) compared to 3.50±1.20 (VIBE), 4.32±0.21 (MAG) and 4.54±0.21 (PSIR) compared to 3.59±0.20 (VIBE) in metastases between 2.5 and 5 cm, 4.09±0.22 (MAG) and 4.50±0.21 (PSIR) compared to 3.41±0.24 (VIBE) in metastases between 1 and 2.5 cm, 4.27±0.15 (MAG) and 4.72±0.12 (PSIR) compared to 3.22±0.17 (VIBE) in metastases between 0.5 and 1 cm, and 4.06±0.27 (MAG) and 4.38±0.18 (PSIR) compared to 2.62±0.27 (VIBE) in metastases below 0.5 cm. Overall, the results were significantly different for VIBE vs. MAG, VIBE vs. PSIR, and MAG vs. PSIR (p < .001) in the variance analysis (Fig 1(B)). Image examples are given in Figs 2 and 3.

### Quantitative evaluation

Mean LLC was 0.35±0.01 for VIBE sequences, and 0.73±0.01 for MAG. The difference was statistically significant (p <0.05). For LCC, the ICC mean-ratings on the individual size level were found to be at least moderate, but also good and excellent with values ranging from.675 to.960. With regards to the model that involved the pooling of all values, ICC mean-ratings were found to be.664 and.873.

**Fig 4:**
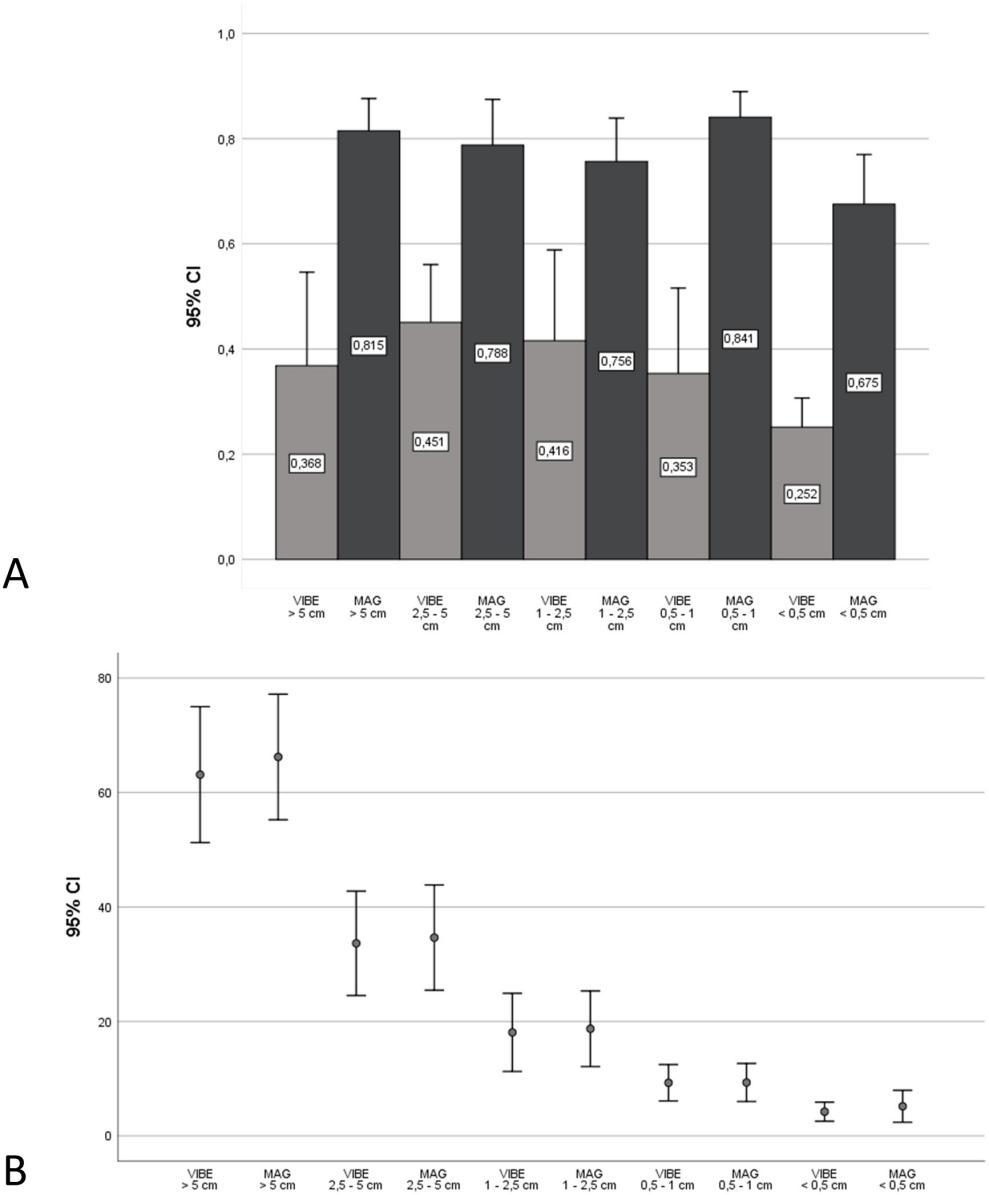
Quantitative comparisons. (A) Mean LLC as measured on VIBE and on magnitude-reconstructed IR (MAG) sequences. The difference was statistically significant (p <0.05). (B) Size measurements revealed a close correlation between the sizes of metastases between both sequence types with slightly larger diameters on PSIR images in comparison to VIBE images. Typically, MAG values were slightly higher than VIBE values. The average difference was 1.30 mm.

Subdivided into the different size groups, LLC on VIBE sequences compared to PSIR was 0.39±0.15 vs. 0.84±0.06 for lesions larger than 5 cm; 0.39±0.02 vs. 0.77±0.02 for lesions between 2.5 and 5 cm; 0.35±0.03 vs. 0.70±0.03 between 1 and 2.5 cm; 0.35±0.03 vs. 0.76±0.02 between 0.5 and 1 cm; and 0.27±0.07 vs. 0.66±0.16 for lesions smaller than 0.5 cm. Due to the small sample size, differences in the size group above 5 cm and below 0.5 cm were not tested for significance. For the other size groups, differences were significant (p < 0.05). Means and SD are given in Fig 4(A).

Size measurements revealed a close correlation between the sizes of metastases between both sequence types with slightly larger diameters on IR images in comparison to VIBE images. Typically, IR values were slightly higher than VIBE values. This difference ranged from.28 to 3.93, with an average difference of 1.30 (see Fig 4(B)). On average, mean lesion size in metastases larger than 5 cm was 61.00±10.35 on VIBE compared to 64.93±9.15 on IR images, 31.91±1.55 compared to 32.74±1.57 in metastases between 2.5 and 5 cm, 15.75±1.05 compared to 16.74±1.05 in metastases between 1 and 2.5 cm, 8.39±0.39 compared to 8.65±0.41 in metastases between 0.5 and 1 cm, and 4.24±1.02 compared to 4.81±1.58 in metastases below 0.5 cm.

**Fig 5:**
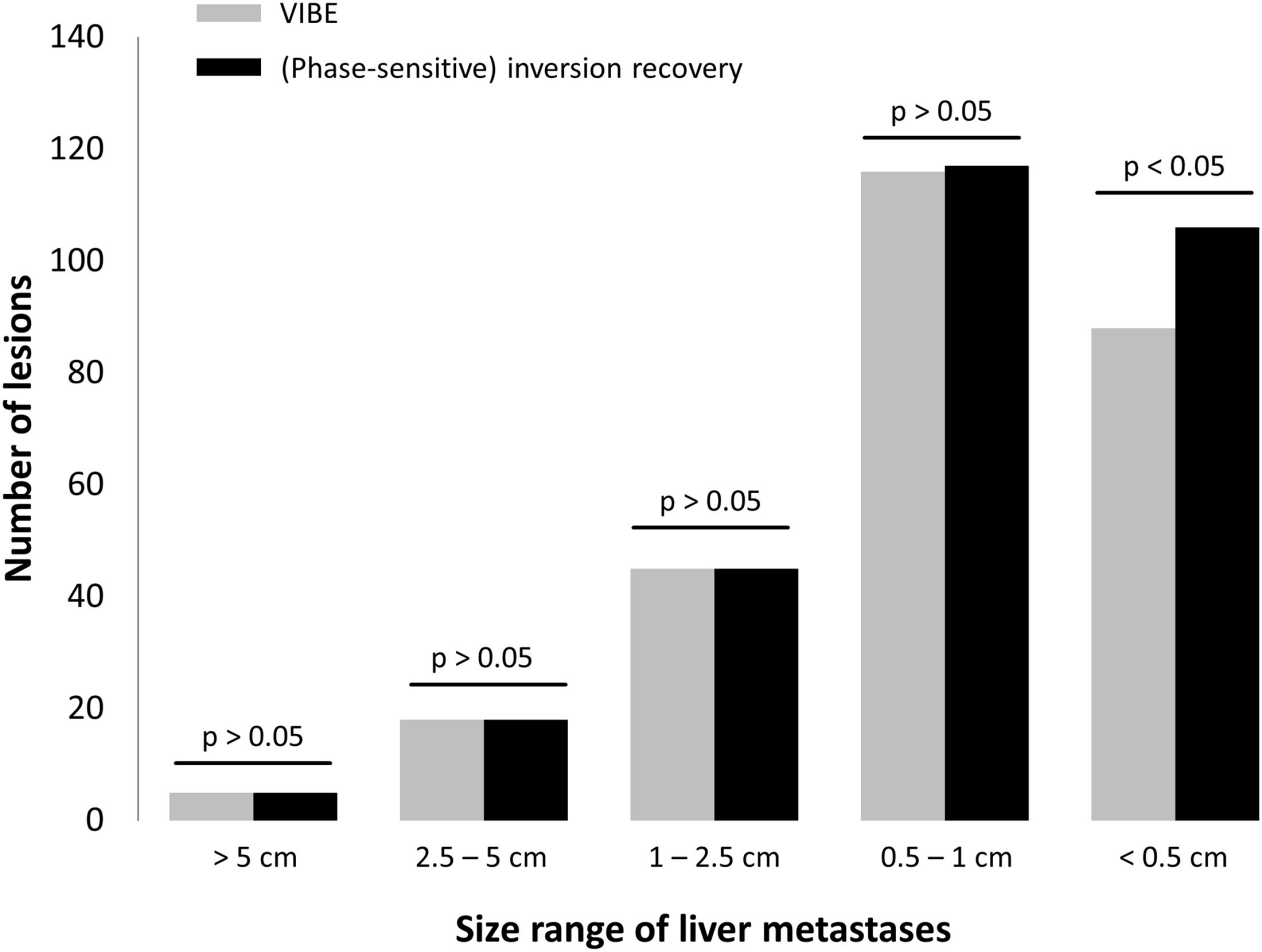
Overall number of metastatic lesions detected in the patient cohort. PSIR images with magnitude reconstruction (black) visualised more lesions with a size between 0.5 and 1 cm and especially below 0.5 cm.

**Fig 6:**
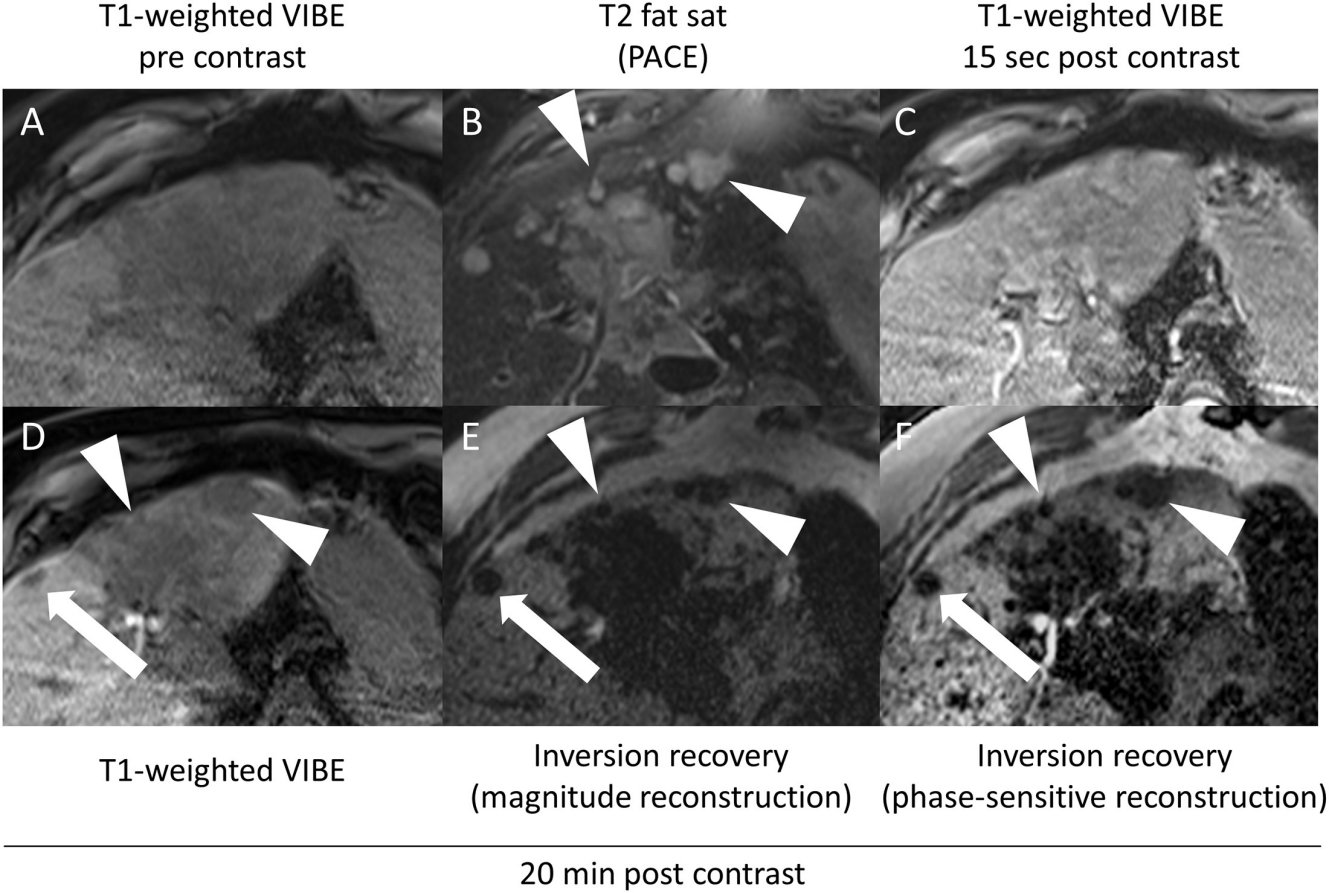
Metastatic tumour in an 80-year-old man with central cholangiocellular carcinoma with metastatic spread. Transverse T1-weighted VIBE (A), and T2 TSE-weighted (B) images. Dynamic VIBE imaging started 15 seconds after intravenous administration of gadoxetate disodium (C). (D) shows VIBE images 20 minutes after contrast agent administration, (E) inversion-recovery with magnitude reconstruction and with phase-sensitive reconstruction (F). The subcapsular lesion is more clearly depicted (white arrow) than on conventional VIBE images. Additionally, several lesions within the liver parenchyma affected by long-standing cholestatic disease can be clearly delineated on inversion recovery images, whereas reduced gadoxetate disodium uptake diminished delineation on conventional VIBE images (arrowheads).

Comparing VIBE and PSIR images, detection was equal for metastases larger than 5 cm (n = 5), between 2.5 and 5 cm (n = 18), and between 1 and 2.5 cm (n = 45). For metastases between 0.5 and 1 cm, an additional lesion was detected on PSIR images (116 on T1 VIBE vs. 117 on PSIR). Regarding the size group smaller than 0.5 cm, according to the above mentioned criteria, PSIR revealed additional liver lesions (n = 18) in 4 patients, of which 15 were detected in one patient (106 vs. 88, Fig 5). This was a statistically significant finding (p <0.05). Additional detected lesions could be correlated to lesions on T2 PACE images. An image example is given in Fig 6.

## Discussion

The contrast-optimised PSIR sequence enabled imaging with a significantly increased contrast between liver metastases and liver parenchyma. Thereby, a higher diagnostic confidence and a higher detection rate of small metastases could be achieved compared to standard VIBE sequences. This is of high clinical importance as most cancer treatment regimens depend on whether the primary tumor is associated with distant metastases [13–15]. Additionally, patients with known hepatic metastases might benefit from a more accurate assessment of tumor burden within the liver, as preoperative imaging is the key in determining the surgical candidacy and approach [13]. Additionally, as metastases in liver parenchyma altered by chemotherapeutic agents can be more difficult to detect, PSIR may offer an improved follow up.

### Detection of liver metastases by clinical T1 weighted MR imaging techniques

Irrespective of their appearance in earlier phases of the dynamic acquisition after contrast agent application, both hypo- and hypervascularised metastases appear hypointense in the hepatobiliary phase as enhancement of the surrounding liver parenchyma is at its maximum, which gives high contrast to the metastatic cells that do not accumulate gadoxetate disodium. Due to this property, hepatobiliary phase imaging is currently the most sensitive time-point for detection of metastases and especially relevant for small lesions [16, 17]. The inherent T1 signal of the metastases in VIBE sequences can, however, reduce imaging contrast between metastases and healthy liver parenchyma. In contrast, on PSIR images, inherent T1 signal of the metastases is suppressed thereby leading to a higher lesion-to-liver contrast ratio.

### (Phase-sensitive) IR based imaging techniques

Different MR sequence techniques can be used to increase the signal ratio between tissues with and without signal enhancement resulting from the T1 shortening effect of contrast agents. In recent years, this issue has been a specific focus in the context of myocardial MR imaging, as a high signal ratio between myocardium and late enhancing myocardial scar is of high clinical importance. For this purpose, specific IR T1-weighted sequences have been developed: A Look-Locker or TI scout is usually performed to determine the optimal TI to minimise signal from the myocardium and thereby maximise contrast between scar tissue and myocardium by increasing the relative signal enhancement resulting from contrast agent accumulation. [7]. Nevertheless, as TI changes over time, the reconstruction of an additional phase-sensitive image was shown to be beneficial, as the imaging contrast in this type of reconstruction is less dependent on an exact determination of the TI [12]. The key difference between conventional IR techniques and phase-corrected or PSIR techniques is the type of the underlying image reconstruction. In the clinical setting, nearly all SE and IR sequences use a magnitude reconstruction. With magnitude reconstruction, the signal of the x and y channels of the coil are combined resulting in a magnitude image which is always positive and an increase in signal to-noise ratio (SNR) of approximately 40%. However, the magnitude image does not take into account the direction of the longitudinal component of the magnetisation. Contrarily, PSIR respects the information from the x and y channels which has several advantages as the technique is less dependent on the overall dose of the contrast agent with regard to patient’s body weight, the time interval between contrast agent administration and image acquisition, and wash-out of the contrast agent from the metastases. This is because PSIR restores the signal polarity of the image by using phase information provided by in the background reference acquisition. The reference image is acquired to estimate the coil field maps and the background phase. Therefore, TI, in general, does not have to be adjusted perfectly and an approximative value can be chosen to suppress the signal. For the assessment of metastases in liver imaging, this is especially of value, as different primary tumours lead to differing structural aspects which may alter the inherent T1 signal.

### Contrast-optimised PSIR imaging for visualisation of hepatic metastases

To perform a direct comparison between PSIR and VIBE sequences, all relevant imaging parameters were matched in this study. In the current study, a TI scout was initially performed to evaluate the optimal TI to suppress or at least minimise signal from liver metastases. Using this approach, the highest T1 contrast between liver metastases and gadoxetate disodium enhancing liver parenchyma in the hepatobiliary phase could be achieved, which was reflected by a significantly higher LLC for the PSIR sequence compared to the VIBE sequence, and eventually led to a higher detection rate of very small metastases in selected patients compared to the standard VIBE sequence. The measured lesion size was higher for the IR sequence compared to the VIBE sequence, which can be explained by the higher LCC and thereby the sharper delineation of the metastases compared to surrounding liver parenchyma.

In patients with cirrhosis or impaired liver function due to chemotherapeutic agents, Gd-EOB-DTPA uptake may be impaired, which accordingly reduces delineation of lesions. Due to the small number of patients with altered liver parenchyma, we did not perform a dedicated evaluation of lesion detectability in these patients, nevertheless, on a patient basis, the PSIR sequence also allowed in these cases an improved delineation of lesions.

### Limitations

Our study is associated with several limitations. First of all, the small sample size prevents drawing conclusions on the expected clinical improvement in detection rate and suffers from bias of selection of patients. Second, we did not subclassify the metastases according to their primary tumour, as this would have led to very small sample sizes. Therefore, the relative frequency of primary tumours included in this study may not represent the frequency of primary tumours in the general population. Additionally, in patients with several lesions, only one clearly definable lesion was included in analysis which inevitably leads to a selection bias. Third, presence of metastatic liver disease was confirmed by histological assessment based on a single lesion in the majority of patients. Therefore, most of the lesions were classified as metastases based on imaging criteria, which may be limited by the absence of DWI, which is considered as a useful sequence for the detection of small lesions. This could lead to false positive and negative results, nevertheless, as the present study was done as a proof of concept, further validation including larger sample sizes could be of value.

## Conclusions

In conclusion, contrast-optimised PSIR improves imaging characteristics of hepatic metastases and may increase detection of small lesions in gadoxetate disodium-enhanced scans compared to T1 gradient-echo VIBE sequences.
